# Multi-Step Hourly Power Consumption Forecasting in a Healthcare Building with Recurrent Neural Networks and Empirical Mode Decomposition

**DOI:** 10.3390/s22103664

**Published:** 2022-05-11

**Authors:** Daniel Fernández-Martínez, Miguel A. Jaramillo-Morán

**Affiliations:** 1 Department of Mechanical, Energetic and Material Engineering, School of Industrial Engineering, University of Extremadura, Avda. Elvas s/n, 06006 Badajoz, Spain; danielfm@unex.es; 2 Department of Electrical Engineering, Electronics and Automation, School of Industrial Engineering, University of Extremadura, Avda. Elvas s/n, 06006 Badajoz, Spain

**Keywords:** times series forecasting, short term prediction of power consumption, multivariate time series, Long Short-Term Memories, Gated Recurrent Units, Empirical Model Decomposition

## Abstract

Short-term forecasting of electric energy consumption has become a critical issue for companies selling and buying electricity because of the fluctuating and rising trend of its price. Forecasting tools based on Artificial Intelligence have proved to provide accurate and reliable prediction, especially Neural Networks, which have been widely used and have become one of the preferred ones. In this work, two of them, Long Short-Term Memories and Gated Recurrent Units, have been used along with a preprocessing algorithm, the Empirical Mode Decomposition, to make up a hybrid model to predict the following 24 hourly consumptions (a whole day ahead) of a hospital. Two different datasets have been used to forecast them: a univariate one in which only consumptions are used and a multivariate one in which other three variables (reactive consumption, temperature, and humidity) have been also used. The results achieved show that the best performances were obtained with the multivariate dataset. In this scenario, the hybrid models (neural network with preprocessing) clearly outperformed the simple ones (only the neural network). Both neural models provided similar performances in all cases. The best results (Mean Absolute Percentage Error: 3.51% and Root Mean Square Error: 55.06) were obtained with the Long Short-Term Memory with preprocessing with the multivariate dataset.

## 1. Introduction

Electric demand forecasting has become a fundamental tool for companies generating, distributing, selling or buying electric energy since it provides them an estimate of the society’s energy needs. This information will help producers to adapt their production and growth policies to social demands, so that the companies’ resources can be optimized to provide a quality supply. It will be also helpful for buyers to plan their purchasing strategies to obtain better prices when buying. To obtain reliable and accurate predictions of electric demand, a detailed description of society’s needs is required, but this knowledge is very difficult to model because many factors can influence it and several predictions are difficult to formulate mathematically. Thus, to provide such predictions, a heuristic method based on numerical information regarding demand and influential factors should be used. In other words, predictions could be provided from historical data of all of these variables. When this information is presented in an orderly fashion, it generates what is known as a time series. Based on this organization of information, three types of demand forecasting processes can be distinguished according to their time horizons: short-, medium-, and long-term.

Short-term forecasting aims at predicting the energy demanded by consumers in the next minutes, hours or days. It is particularly useful for power generation companies to schedule production operations to fit the market demands, for distribution companies to properly adjust the distribution grid to that demand and, finally, for buyers to plan their purchasing strategies to obtain the best prices. Medium-term predictions seek an estimation of the energy consumers will demand in the coming weeks or months. They are useful for network maintenance planning or fuel purchase management in power generation plants. They may also help dealers in the energy markets to define business management policies and purchase bids. Finally, long-term prediction aims at forecasting the overall yearly demand or load peaks for the coming years in order to plan expansion strategies for both power plants and transmission and distribution grids.

Nowadays, short-term forecasting has become critical for companies dealing with electric energy because of the fluctuating and rising trend of electricity prices. This has forced them to schedule accurate electric energy purchasing plans to minimize their acquisition costs. Companies buying electric energy for themselves or for other consumers whose energy purchases they manage need accurate and reliable forecasts to make the most cost-effective purchase offers. Therefore, they need tools capable of providing reliable power demand forecasts to manage their purchasing strategies and obtain the most favorable prices. Thus, accurate predictions of hourly and daily demands should be provided. This fact requires the development of reliable forecasting models adapted to the nature and needs of each consumer. Different models should be developed for different customers with different consumption patterns and energy needs. Thus, besides research devoted to predicting overall hourly or daily demand [1,2,3,4,5], other studies have focused on specific consumers [6,7,8,9,10], and different forecasting tools have been proposed to obtain reliable demand predictions. These predictions may have a one-step-ahead horizon or a multi-step one. Despite that this last structure seems the most advisable option, there are few researchers studying it [1,8,10,11,12], a fact that is hardly surprising considering that it represents a more challenging problem. That is why the models studied in this work will provide hourly predictions of the electric energy that a specific consumer will demand in the following 24 h, i.e., 24 predictions will be provided all at once. Thus, this customer will have an estimation of the daily consumption profile in advance for better planning his/her purchase offers.

On the other hand, as short-term demand is highly influenced by variables different from its time evolution such as meteorological or social factors, information regarding those exogenous variables may be also included in the forecasting models along with data describing the time evolution of demand. When these kinds of variables are added to the forecasting model, we obtain what is known as multivariate time series, in contrast with the univariate ones, in which only historical data of that to be predicted are used. Usually, the multivariate option is preferred because it is usually assumed that the most information provided to the forecasting model, the higher the accuracy obtained. Thus, only few works have been devoted to the univariate option [13,14].

As electric energy and variables influencing it have a time-evolving behavior, they may be analyzed as time series, and, therefore, tools usually used to carry out predictions in this field can be applied to obtain those predictions. Those techniques may be roughly classified into two categories [15]: statistical methods and those based on Artificial Intelligence. The former represents what can be considered as classical tools, since they were the first to be applied to times series forecasting. Thus, tools such as linear regressions have been used to forecast short-term loads [16]. More elaborate tools such as Kalman filters [17] have been also used to forecast electric loads. Nevertheless, the most popular method is the Autoregressive Integrated Moving Average (ARIMA) [18], which was specifically designed to predict time series. Although it has been widely used for this task [19,20,21], it has serious problems when dealing with strongly nonlinear ones, such as those related to the electric load, energy demand or price evolution because of its linear structure. Thus, it needs to be combined with other data processing methods to try to improve its performance. In this way, ARIMA has been used with a Fourier Series Expansion and Particle Swarm Optimization (PSO) to improve a seasonal ARIMA to forecast electricity consumption [22] or with Autoregressive Conditional Heteroscedasticity (ARCH) to forecast CO_2_ emissions in Europe [23].

Although these hybrid models clearly outperformed the basic ARIMA, new forecasting tools have been developed to forecast times series with the aim of improving the performances provided by the statistical models. They are mainly based on Artificial Intelligence, defining the second type of forecasting tools mentioned above [24]. They are especially well suited to deal with time series with nonlinear behavior, where classical statistical methods fail to provide accurate predictions. In fact, they clearly outperform classical ones: in [15], several forecasting models were tested, and those based on Artificial Intelligence clearly outperformed statistical ones in electricity price forecasting. There are several of those models that can be used for time series forecasting, such as Random Forest (RF), Gradient Boosting (GB), and Extreme Gradient Boosting (XGBoost) [25] or SVR [26]. Nevertheless, Neural Networks (NN) have been widely used to forecast variables related to the economy or energy and have become one of the most popular forecasting methods in those fields. Thus, they have been used to forecast electric power transactions [27], natural gas demand [28], electric energy consumption [29], stock market variables [30,31], electricity prices [32] or CO_2_ emission allowance prices [33,34]. Many of these applications, in fact, all those cited above, use a very simple neural model: Multilayer Perceptron (MLP), a classical neural model with a very simple structure. It has become one of the most popular neural models because of its simplicity and good performance both in classification and regression problems. Nevertheless, it is not the only neural model that can be used in time series forecasting. The development of new complex neural structures known as Deep Learning neural networks has attracted the attention of researchers to use them as time series forecasting tools [15]. Their defining characteristic is that they are made up of a large number of layers and neurons (processing elements) within each one. One of those structures has focused that attention, Long Short-Term Memory (LSTM), which was originally developed to process a high amount of data with a strong time dependency and has been used to process written text or speech, providing very accurate results. Thus, it seems logical to assume that they can also provide accurate predictions in time series forecasting, as a great amount of data, with strong time dependency, is usually used to obtain future values. Therefore, they have been used to predict electric energy load [1,4,5,14,35,36,37,38], electricity prices [29] energy production in photovoltaic plants [39], consumption in residential areas [7] and buildings [8,9] or CO_2_ emission allowance prices [40].

Although LSTMs are able to provide accurate predictions [1,5,9,35,37,38], they have been used along with other tools to improve performance, in the same way as was done with the statistical tools. Thus, in [2], the structure was combined with a Convolutional Neural Network (CNN, another class of Deep Learning networks) to provide short-term electric load forecasting, while in [39], this same structure was used to forecast power generation in a photovoltaic plant; in [41] it was combined with a CNN and an ARIMA to predict future prices of CO_2_ allowance prices in the European Union, and in [14], a Genetic Algorithm was used to optimize an LSTM.

Another technique that may be used to improve performance is to preprocess the dataset to provide a modified version of the time series that may be more easily forecasted [42]. A widely used option when forecasting energy-related time series is the decomposition of the original dataset into several sub-series that can be independently forecasted and then summed to provide a prediction of the original time series. The aim is to obtain new time series that may be more easily forecasted, as they are supposed to have a more or less periodical behavior. This assumption is based on the fact that energy-related variables are closely related and influenced by social and weather factors that have a certain periodical behavior. Thus, those subseries should be related to specific frequencies embedded in the overall behavior of the time series, and, consequently, can be more accurately predicted. One of those techniques is the Empirical Mode Decomposition (EMD), which splits a time series into several subseries, each one with a proper oscillatory behavior. As the whole process has a heuristic behavior, this algorithm suffers from a lack of mathematical support. The Variational Mode Decomposition has been developed to overcome this problem. Both algorithms have been applied to different forecasting tools such as ARIMA [43], MLP [40], CNN [44], LSTM [4,5,40], SVR [26] or spiking neurons [45]. All of these studies showed that the models with preprocessing outperformed those without it, a fact that proves the beneficial effect of including preprocessing into the forecasting models. Thus, it has become a fundamental step in the forecasting process, because, as the aforementioned works have proved, it has improved the performance of the forecasting tools when properly selected and applied to the time series to be predicted.

As pointed out above, short-term forecasting of electric energy demand has become a key issue for companies purchasing electricity because of the rising and fluctuation trend of prices, which forces them to continuously adjust their purchasing bids to the fast-changing evolution of those prices; not only is the short-term forecasting of the overall energy demand necessary, but also a more specific one focused on the electric load of specific companies, institutions or buildings is needed, as those medium-small consumers also demand an accurate prediction of their energy needs to optimize their electricity purchases. Several works have been devoted to providing reliable forecasting tools for those customers [6,7,8,9,10]. Nevertheless, there are not many papers devoted to forecast consumption of those specific consumers because data needed to carry out those tasks must be actual measurements provided by specific equipment and belong to consumers who are not always willing to share them with time series researchers. Therefore, new contributions proposing different forecasting tools processing actual information should be encouraged to provide an ensemble of forecasting tools as large as possible that can be applied to different customer needs in order to find out the most effective one for each particular case. There are many forecasting structures that may be applied to predict short-term consumption, as pointed out above, and a number of them are complex hybrid models that have been able to improve the accuracy of more simple tools. Nevertheless, although accurate and reliable, they are hard to use and need complex simulation programs. In this work, a relatively simple hybrid model is developed, which is made up of Deep Learning neural networks and a previous preprocessing stage based on EMD. It will be used to carry out hourly consumption predictions of a public facility: a hospital. The aim is to provide a relatively simple forecasting structure based on computational models that are directly available in programming environments usually used in Artificial Intelligence, so that they may be easily implemented. Deep Learning neural networks have been selected as the forecasting tools because, as pointed out above, they are specifically well suited to deal with huge amounts of data as is the case of the problem in hand. In fact, data different from electricity consumption, which are directly related to it, will be processed along with it to obtain predictions (multivariate model). These two facts define a problem of prediction implemented in a data fusion environment (lots of data of different nature used altogether), which aims at providing consumption needs in advance to facilitate information to small–medium consumers to optimize their energy purchasing strategies.

It will be proved in this work that a hybrid forecasting model made up of a Deep Learning neural network and a preprocessing tool processing a multivariate dataset will be able to provide accurate and reliable prediction of the following 24-hourly consumption, in other words, the hourly consumption profile of the following day. It will be also proved that their forecasting errors are also significantly lower than those provided by the models without preprocessing or working with a univariate dataset (only consumptions are used to obtain predictions).

The remaining of the paper is organized as follows. In Section 2, the forecasting tools and the preprocessing algorithms to be used are described. In Section 3, the overall forecasting process is described, while in Section 4, the dataset to be predicted is studied. In Section 5, the forecasting structures used are analyzed; the results obtained are presented and discussed in Section 6. Finally, in Section 7, the conclusions are presented.

## 2. Materials and Methods

### 2.1. Deep Learning Neural Networks for Time Series Forecasting

Neural Networks are a set of data processing models that define a field of Artificial Intelligence that try to reproduce the behavior of the human brain in some practical applications by means of processing structures that try to mimic its organization. Those models are developed from a basic processing element that represents the basic element of the brain: the neuron. Many them are arranged into superimposed layers to define a neural network whose structure should be adapted to the problem to be solved. They have proved to succeed in solving problems closely related to human abilities where other tools fail to provide reliable answers. They have been able to provide better performances than other classical tools in fields such as time series forecasting, data classification, image processing, object identification or pattern recognition. Moreover, new problems that could not be previously addressed with those classical tools can now be tackled with neural structures specifically developed to dealt with them. Thus, very complex neural structures comprised of thousands, and even millions, of neurons have been developed to recognize speech or text. For the problem at hand, short-term load forecasting, Neural Networks have provided excellent performance, so that they have become one of the most widely used tools for this task. One of those models is a very simple structure closely related to the basic biological structure of Neural Networks: the Multilayer Perceptron (MLP) [46]. It was one of the first models developed and, despite its simple structure, it has been able to provide accurate predictions in the field of time series forecasting. Nevertheless, other neural models have also attracted the attention of researchers as they were developed to deal with a large amount of data with temporal dependencies, as is the case of short-term load forecasting. One of those models is the Long Short-Term Memory (LSTM) model, which has been widely used in this field, as pointed out above.

#### 2.1.1. Long Short-Term Memories

LSTM [47] is one of the neural models that perform what is known as Deep Learning because of its high number of layers and neurons. Thus, it has a multilayer structure where information flows from the input layer, which receives the data to be processed, through several hidden layers to the output one, providing the network response. Each layer is made up of several processing units (cellular blocks) that may include one or several neurons. Each neuron receives all the outputs of those in the preceding layer along with feedback from the other ones in its own layer. All this information is combined with a sort of “memory” of past states of the neuron to compute its response. Thus, each cellular block and therefore each neuron inside it, processes three different types of data: the outputs of the preceding layer (new data), feedbacks from neurons in the same layer, and past information stored in it. The neuron will determine which of them will be used to provide its response. To do that, each block has control gates that determine which information will be used to provide the neurons’ outputs and whether this output exits from it. There are only one set of gates to control all the neurons inside each block. Therefore, each neuron will receive data from neurons in the previous layer, which is added to those from the neurons in the same one to be processed by a saturating function, σ, which uses the hyperbolic tangent. Thus, the equation describing the neuron input is [48]

(1) $$z_{j}=\sigma \;\left(W_{z}\cdot \left[x^{t},\;h^{t-1}\right]+b_{z}\right),$$

where ***x****^t^* represents the data from the previous layer, ***h***^*t*−1^ represents those from feedback, and *b_z_* is a bias weight. ***W_z_*** is a weight matrix defining the strength with which each datum enters the neuron.

Then, the neuron will combine this datum with information stored inside it, a sort of “memory”, which represents an inner state recording past data received by the neuron up to this moment. This process is described by [48]:

(2) $$c_{j}^{t}=i_{j\;}\cdot \;z_{j}+f_{j\;}\cdot \;c_{j}^{t-1},\;$$

where $c_{j}^{t}$ is the neuron’s state, *i_j_* (input gate) represents a control gate that determines whether the new information entering the neuron will be added to the neural “memory”, and *f_j_* (forget gate) is another one that determines whether that memory is forgotten or considered to define a new neural state.

Finally, another gate, *o_j_* (output), will determine whether the neuron will provide an output, which will be the neural inner state previously processed by another hyperbolic tangent [48]:

(3) $$y_{j}=o_{j\;}\cdot \;\sigma \left(c_{j}^{t}\right)$$

The control gates used in the above expressions are defined by functions such as the neural input (1) [48]:

(4) $$i_{j}=\sigma \;\left(W_{i}\cdot \left[x^{t},\;h^{t-1}\right]+b_{i}\right),$$

(5) $$f_{j}=\sigma \;\left(W_{f}\cdot \left[x^{t},\;h^{t-1}\right]+b_{f}\right),$$

(6) $$o_{j}=\sigma \;\left(W_{o}\cdot \left[x^{t},\;h^{t-1}\right]+b_{o}\right).$$

In these expressions, σ() is a sigmoid function, as control gates determine whether information is considered; it should have a value between 0 and 1. ***W_i_***, ***W_f_***, and ***W_o_*** represent weight matrices, and *b_o_*, *b_f_,* and *b_o_* are bias weights. Thus, these gates will allow data to procced based on the inputs the neuron receives, both from the previous layer and from the feedback form the same one. When a cellular block is made up of more than one neuron, all blocks will have the same control gates, although each one will have different inputs (1), states (2), and outputs (3).

As LSTMs are neural networks, they should be trained to learn to carry out a certain task. To do that, the available data should be split into two sets: one for training and the other to validate the proposed model. The datasets do not have the same length because it is assumed that if more data are used to train a model, the better it will capture the behavior of the system defined by the data. Percentages between 60 and 40% (training–validation) and 80 and 20% are usually used. Although each neural model has its own training algorithm, there is one that has been used by several of them: the one known as Backpropagation [46]. It was originally developed for the MLP and has been adapted for other models, such as LSTM. Basically, this algorithm measures the difference between the network output when training data are presented to the input and the desired output, calculating a global error whose value should be minimized by properly adjusting the neural input weights. Then, the error is backpropagated from output to input to adapt those weights. This basic algorithm is adapted to the structure of the LSTM to consider the effects of feedback in the network structure. Thus, two modifications are included to deal with the different nature of gates and activation functions [49]: the truncated Backpropagation Through Time (BPTT), which is used to adjust the weights of the output units and output gates, and the Real-Time Recurrent Learning (RTRL), which is applied to adapt the weights of cell inputs, input gates, and forget gates.

#### 2.1.2. Gated Recurrent Unit

The Gated Recurrent Unit (GRU) [50] represents a simplification of the structure of the LSTM that was originally developed for speech recognition [51]. Two simplifications were considered. The first is the removal of the activation function providing the neural output, which now is the neuron’s inner state [51]:

(7) $$y_{j}=o_{j\;}\cdot \;c_{j}^{t}$$

The second simplification is the elimination of the forget gate, which is replaced by the opposite of the input gate, so that the inner state update will be carried out by adding new information and removing that stored by ignoring new data and retaining that in “memory”. The new equation defining the inner state upgrade is [51]

(8) $$c_{j}^{t}=i_{j\;}\cdot \;z_{j}+\left(1-i_{j\;}\right)\cdot \;c_{j}^{t-1}.$$

As the values of *i_j_* ranges from 0 to 1, the contribution of new and old information will not be mutually exclusive.

GRUs have been used for short-term load forecasting [52,53,54]. They have been able to provide better performance than LSTM in some cases [54].

### 2.2. Data Preprocessing: Empirical Mode Decomposition

Although Artificial Intelligence tools are able to provide accurate and reliable predictions when forecasting nonlinear time series, their performance may be improved if input data are adequately preprocessed to obtain a new dataset that could be more easily predicted. This process may be a simple statistical treatment to try to remove possible erroneous data or to identify some statistical properties or very sophisticated procedures that change the time series to obtain an equivalent representation that could be more easily predicted. Therefore, the preprocessing algorithms to be used will depend on the nature and behavior of the times series to be processed. Often, time series have an oscillatory behavior that suggest that if it could be split into several subseries, each one related to a certain frequency, the forecasting accuracy could be improved, as those subseries should be more easily forecasted. Therefore, a good strategy to carry out the preprocessing process could be to identify and extract periodical components that could be more easily forecasted. Nevertheless, these components are hardly ever represented by an isolated frequency. Instead, sets of close related ones are observed. To deal with such a data structure, an empirical tool has been developed that provides a decomposition of a time series into a set of subseries, each one with a proper oscillatory behavior more or less related to a certain frequency. This tool is the Empirical Mode Decomposition (EMD) [55,56], and each one of the subseries it provides is known as an Intrinsic Mode Function (IMF). These IMFs are not periodical functions but time series that accomplish this with two properties [56]:

- The number of local minima and maxima must be equal or differ only by one.
- Its mean value must be zero.

The first condition means that one only extreme point can appear between two consecutive zero-crossing points, while the second means that the series is stationary.

Thus, a time series with an oscillatory behavior can be decomposed into the sum of IMFs and a residue:

(9) $$x\left(t\right)=\underset{n}{\sum }x_{n}\left(t\right)+r\left(t\right).$$

The process of obtaining the IMFs works as follows [56].

Step 1: All maxima and minima points of the time series will be identified and selected.

Step 2: Two envelops, upper and lower, will be defined with both maxima and minima by means of cubic splines. Then, their mean series, *m*_1_, will be obtained.

Step 3: A first candidate to be an IMF will be obtained by subtracting *m*_1_ from *x(t)*:

(10) $$c_{1}\left(t\right)=x\left(t\right)-m_{1}\left(t\right).$$

Step 4: In the case that $\;m_{1}$ fulfills the two aforementioned conditions, it will be considered the first IMF ($x_{1}\left(t\right)=c_{1}\left(t\right))$ and the process will jump to the following step. Otherwise, it will be considered a new time series to be analyzed, and steps 1–3 will be repeated until an IMF is obtained.

Step 5: Once this first IMF is identified, it will be subtracted from the original time series to obtain a new one:

(11) $$d_{1}=x\left(t\right)-x_{1}\left(t\right).$$

Step 6: Steps 1–5 will be repeated with this new series until no new IMF is obtained.

Once the process stops, the last IMF is subtracted from the time series from which it was obtained to provide the residual $d_{n}=r\left(t\right)$.

This algorithm needs two stop conditions: one for the process of obtaining a new IMF (steps 1 to 3) and the other for the whole process. The first one should be accomplished when an IMF candidate fulfills the two required conditions or a prefixed value for the IMF candidate variance is achieved. The second stop condition will be reached when the residue $d_{n}$ is a constant, has a constant slope, or contains one only extreme.

Nevertheless, this process has the drawback that the same frequency may appear in more than one IMF, or one IMF may be made up with quite different frequencies. To overcome this problem, a modification to this algorithm has been proposed: the Ensemble Empirical Mode Decomposition (EEMD) [57]. In this new version, different profiles of white noise are added to the time series so that several corrupted series are obtained. They will be independently treated by the EMD algorithm to provide their corresponding decompositions (Equation (9)). Then, the mean value of the equivalent IMFs of each one is calculated. In this way, a new decomposition of the original time series is obtained as follows [58]:

(12) $$x\left(t\right)=\underset{n}{\sum }{\bar{x}}_{n}\left(t\right)+\bar{r}\left(t\right).$$

In this expression, ${\bar{x}}_{n}\left(t\right)$ represents the mean value of equivalent IMFs of the decomposed representation of each corrupted times series and $\bar{r}\left(t\right)$ the corresponding mean value of the residues.

This new version of the basic EMD has the problem that the number of IMFs for each corrupted time series could be different in some cases. To overcome this issue, a modification of this algorithm has been proposed in which the process of obtaining each IMF is carried out in parallel. This means that the first IMF is obtained for each corrupted series and then their mean value calculated. It will be considered as the first IMF of the original time series: ${\bar{x}}_{1}\left(t\right)$. Now, it will be subtracted from that original times series and a residue will be obtained, which will be considered the new series to be decomposed. The whole process is repeated until the last IMF (${\bar{x}}_{n}\left(t\right))$ is obtained and the overall residue calculated. This new version is known as Complete Ensemble Empirical Mode Decomposition (CEEMD) [58,59].

## 3. Forecasting Model Structure

In this work, the prediction of the electric energy demand of a healthcare building in the following 24 h, i.e., the energy it will probably need in the following day, will be obtained. The forecasting model proposed in this work will use Deep Learning neural networks as predictors to provide 24-h future data of consumption. Two neural models will be independently used, LSTM [47,48] and GRU [50,51], to test their performances and to find out which one provides the more accurate predictions. They will process a set of past data (inputs to the forecasting model) of the hourly electric consumption of this public facility along with past data of weather variables (temperature and humidity) and reactive energy consumptions. Their outputs will be 24 predictions of consumption (the estimated hourly demands of the following day).

In order to improve the performance of the forecasting models, all available data will be preprocessed before been passed to the prediction tools to provide datasets that could be more easily forecasted. This process will be divided into two stages. In the first one, the time series representing those variables will be statistically treated to detect and correct anomalous data. They will then be scaled to provide the neural models with datasets that are easier to process. These treatments are a common practice in time series forecasting that aim at providing new versions of the datasets that could be more easily treated. In the second stage, an algorithm will decompose the statistically treated time series into a set of subseries with a more or less uniform periodic behavior. This process will be carried out by the EMD [55,56] or the CEEMD [58], or a modification of this. It is expected that these new subseries will be more easily processed by the LSTM and the GRU, so that more accurate predictions can be obtained. The predictions of those subseries will be subsequently added to obtain the consumption predictions (which must be rescaled to obtain their actual values). The combination of a neural network (LSTM or GRU) with a decomposition algorithm (EMD or CMD) defines the hybrid forecasting model proposed in this work.

**Figure 1:**
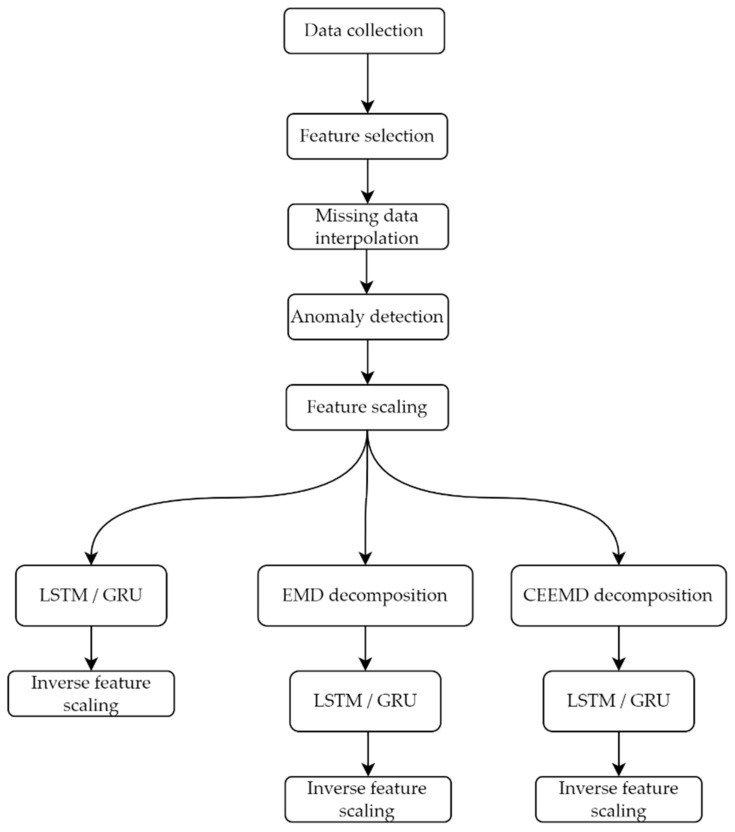
Computational workflow of the whole forecasting process.

The whole process of consumption forecasting carried out in this work is described in Figure 1 for better understanding.

## 4. Data Acquisition and Preprocessing

### 4.1. Data Description and Feature Selection

**Figure 2:**
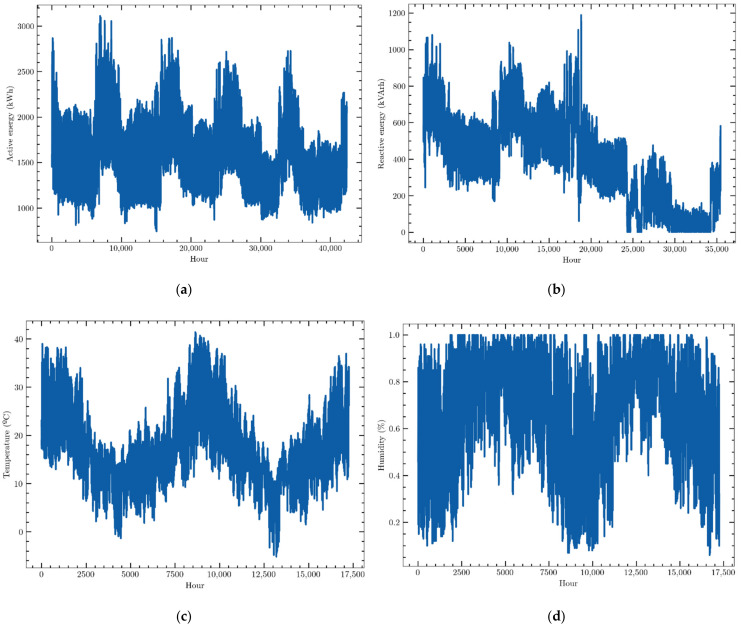
Data sets of (**a**) hourly active electric energy consumption, (**b**) hourly reactive energy consumption, (**c**) hourly external temperature, (**d**) hourly relative humidity.

The data used in this work are hourly consumption and weather variables provided by an electric energy trading and management company, Emececuadrado, based in Badajoz, a city in the southwest of Spain. The demand profile of a hospital is described. These data were collected by a SCADA (Supervisory Control and Data Acquisition) system that monitored the consumption of that customer. They were provided in JSON format, a simple text format for data exchange widely used in software development. Slightly more than 40,000 data points of active electric energy consumption, measured in kWh, were recorded from 1 September 2016 to 1 July 2021 and organized as a time series. They are presented in Figure 2a. They show a clear annual seasonal behavior, with consumption peaks during extreme weather months, that is, during January and February and, above all, during summer months. It may be also seen that the consumption profile has not suffered the effects of the COVID-19 pandemic as no significant modification of its profile may be detected. This means that this fact must not be taken into account in the forecasting models.

Three other variables closely related to active electric energy consumption have been recorded and organized as a time series, which will be used by the forecasting models to try to improve the prediction accuracy:

- Reactive energy: reactive electrical energy consumed by the customer, i.e., consumption measured in kVArh in 1-h intervals (Figure 2b).
- Outside temperature: ambient temperature outside the building, i.e., the external temperature measured in °C every hour (Figure 2c).
- Outdoor humidity: relative humidity of the air outside the building. Data were collected every hour and were measured as a percentage (Figure 2d).

The first 5000 data points of reactive energy consumption were not recorded although they were provided by the measurement system, while data of weather variables started to be measured and recorded on 10 July 2019. These facts suggest analyzing two different case studies to forecast hourly consumption: a univariate prediction scenario that takes into account only the whole dataset of active energy consumption and another multivariate one that uses the four time series, but whose data ranges from 10 July 2019 to the end of the series.

Before using the four datasets, it would be advisable to check whether the exogenous variables (those different from that to be predicted) are related to the energy consumption. This can be done by analyzing the correlations between them. It may be said that two variables are correlated when their time evolution follows more or less the same behavior.

When several variables are to be used and their correlations provided, the correlations between each pair of variables may be arranged into a matrix. The most common of these structures is the Pearson correlation matrix that provides a measure of linear relationships between those variables. It is defined as follows:

(13) $$\rho_{x,y}=\frac{cov\left(x,y\right)}{\sigma_{x}\sigma_{y}}$$

The values provided by this expression range from −1 to 1, so that those coefficients close to 1 define a direct linear correlation between the two variables analyzed, while those close to 0 point to the absence of correlation between them. On the other hand, values close to −1 show an inverse linear correlation, that is to say, the two variables have opposite behavior: when one increases the other decreases.

The correlation matrix of the four variables is presented in Table 1. Only correlations between energy consumption (active energy) and the other three variables are of interest for this work. As shown in this table, correlations are not strong although they all have values higher than 0.5. Thus, it seems reasonable to use those exogenous variables along with consumption to carry out forecasting, at least to check whether their inclusion improves accuracy or not. It is noticeable that correlation with humidity is negative, which points to an inverse correlation between them.

### 4.2. Data Preprocessing

As pointed out above, preprocessing is a first stage in the process of time series forecasting in which available data are treated to remove anomalous vales and to provide a version of the times series that can be more easily forecasted. In this work, three preprocessing stages have been applied: anomalous data removal, rescaling, and EMD decomposition. The first aims at detecting and correcting missing data and outliers. The second compresses the range of values of the datasets to provide a homogeneous distribution that facilitates their processing by the neural models. Finally, the third stage splits the datasets into subseries that can be more easily forecasted. These three stages will be described in detail in the following subsections.

#### 4.2.1. Missing Data and Outliers

It is common in time series of data recorded from real-world processes to lose some of them [60], as more or less wide data intervals and others as isolated points. Although there are models that allow working with these datasets, Deep Learning models are especially sensitive to this phenomenon, thus correcting it is a crucial step to obtain accurate predictions.

For the case of missing intervals of data, a first option could be to rearrange the available data by discarding the missing ones. Although this option may be suitable for classification problems, it may not be appropriate for time series forecasting since time dependency of the data on both sides of the missing interval is broken. For small enough intervals of missing data in time series with a certain periodic behavior, an efficient alternative is to interpolate the missing data with values obtained from a time interval with a behavior similar to that of the missing data.

The case of isolated data is easier to deal with, and a good strategy could be to replace them by linear interpolation between backward and forward data. In this work, since all the missing data in the series studied were isolated, each data point was replaced by the mean value of the datum preceding it and that following it. It is worth noting that very few data were missing in the four time series used.

The treatment of outliers is easier, as they are usually abnormal isolated values that are related to erroneous measurements. They can be removed by replacing them with the mean of the surrounding data. It was not necessary to apply this process to the times series used in this work since they did not present this kind of abnormal data.

#### 4.2.2. Data Scaling

Normalization of the values of a time series is often useful and sometimes necessary when using some Machine Learning algorithms such as Artificial Neural Networks. This is because they use the gradient descent algorithm to train their processing elements (neurons in the case of neural networks), and the presence of data with different ranges of values can affect the convergence of the method. Normalization consists of rescaling the values of the original data set into a more compact interval maintaining the original data distribution. This new interval is usually (0, 1) or (−1, 1). This process is especially necessary in the case of multivariate time series forecasting, where datasets of different natures with different ranges of values are used to predict future values of one of them, as is the case in this work.

As in this work all the data are higher than 0, the four times series used have been normalized to the (0, 1) interval. Because of the absence of outliers in the datasets, the max-mis normalization was used. Thus, the new datasets were obtained with the following expression:

(14) $$y=\frac{x-x_{min}}{x_{max}-x_{min}}$$

where x is the value to be normalized, and $x_{min}$ and $x_{max}$ are the respective minimum and maximum values of the series.

#### 4.2.3. Empirical Mode Decomposition

**Figure 3:**
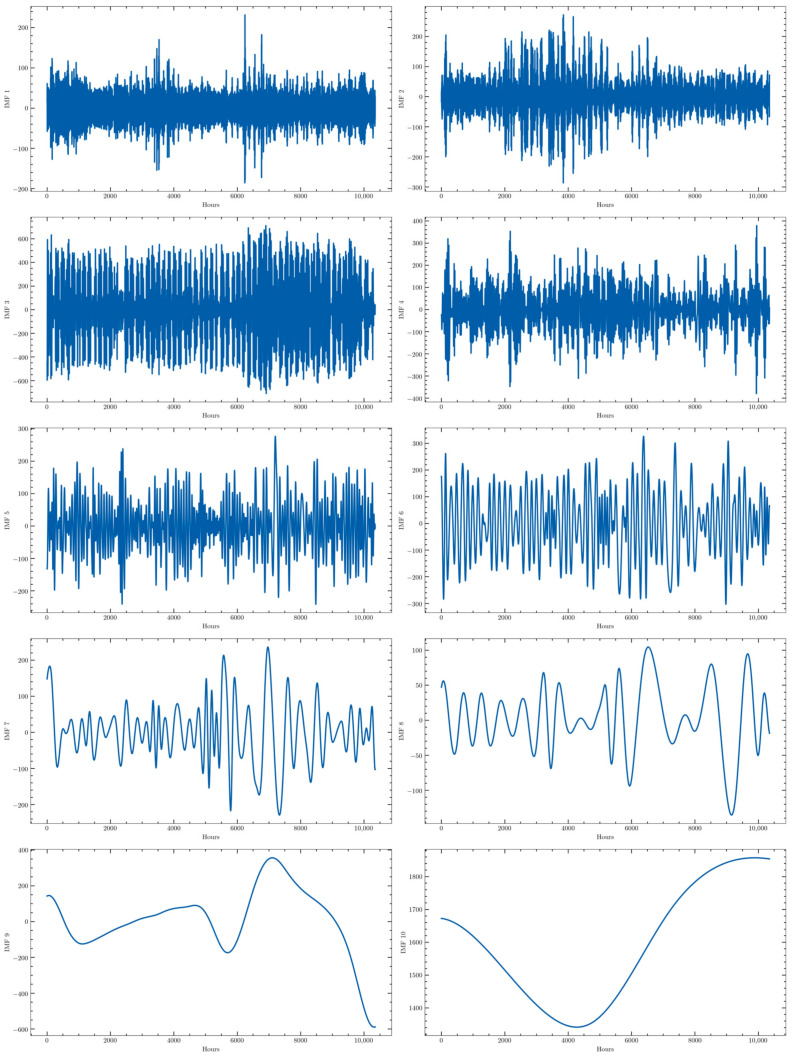
CEEMD of the hourly active energy demand of one of the training data sets (from 10 July 2019 to 13 September 2020).

The last step in the preprocessing stage carried out in this work is the decomposition of the original time series into several subseries with a more or less periodical behavior that could be more easily forecasted. This process has been carried out by applying the Empirical Mode Decomposition (EMD) algorithm to the four time series. This algorithm automatically decomposes the time series into several stationary Intrinsic Mode Functions (IMF), whose number is determined by the algorithm itself. For the time series used in this work, 10 of such subseries were provided. They all are stationary as required by the algorithm. For the sake of comparison, a modified version of this algorithm has been also tested: the Complete Ensemble Empirical Mode Decomposition (CEEMD). It represents an optimization of a modification of the original EMD algorithm, the Ensemble Empirical Mode Decomposition (EEMD), which was originally proposed to avoid the presence of the same frequencies in different IMFs. They will be tested to determine whether this modification actually improves the prediction accuracy. The decomposition obtained with the CEEMD for one of the training subsets (from 10 July 2019 to 13 September 2020) of the consumption time series is presented in Figure 3 as an example.

## 5. Forecasting Structures

**Figure 4:**
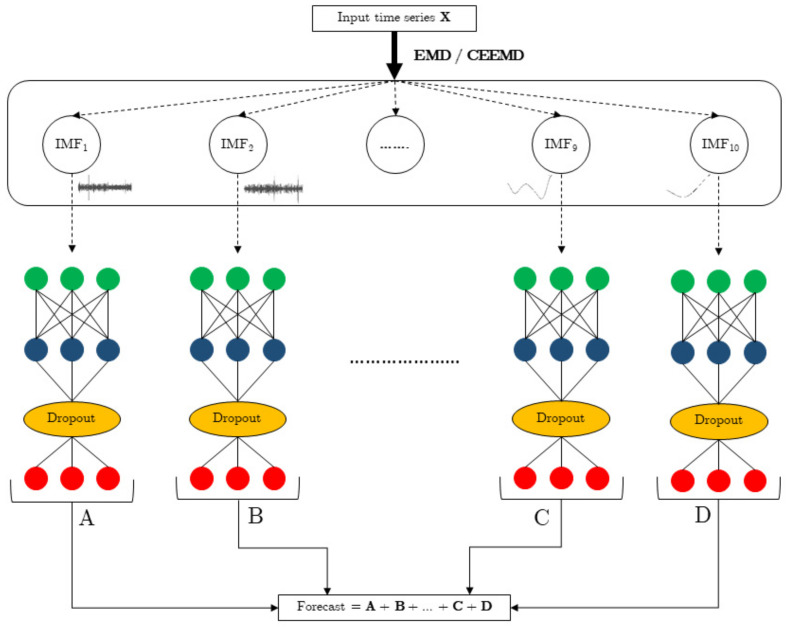
CEEMD–RNN strategy.

The forecasting model proposed in this work is made up of two stages: the first carries out a decomposition of the time series to be processed into a set of IMFs provided by the EMD or the CEEMD algorithms, while the second performs the forecasting process with an LSTM or a GRU neural model. This last process is independently applied to each one of the IMFs obtained. Therefore, there will be as many neural networks as IMFs provided by the EMD algorithm. The same neural model was used for all EMDs. Once all IMFs are processed, the predictions obtained will be added to obtain the corresponding consumption prediction. The structure of this forecasting model may be seen in Figure 4.

As two neural networks (LSTM and GRU) are tested along with two decomposition algorithms (EMD and CEEMD), four forecasting models will be tested, each one with the same inner structure. Both EMD and CEEMD provided 10 IMFs.

When defining the network structure, it is essential to determine the number of layers and processing elements that provide the best performance. As both LSTM and GRU are made up of processing blocks that can have one or several neurons, it must be determined how many neurons each one will have. For the sake of simplicity, one neuron per block was assumed. For the same reason, both networks were defined with one only hidden layer and one output one. Models with two hidden layers were also tested, but performance did not improve and actually worsened. This fact is not surprising, as it is a well-known fact that neural networks with too many neurons tend to provide worse accuracy since they tend to learn the pattern presented to them instead of learning the inner behavior of those patters, i.e., they lose the ability to generalize the acquired knowledge, an effect known as overfitting.

Therefore, the neural models defined have an input layer (the data to be processed), a hidden one (an LSTM or a GRU), and one output layer, and as is usual when working with these neural models, a fully connected MLP with linear activation functions. The aim of this last one is to transform the LSTM or GRU response into the format of the data processed.

Different numbers of neurons in the hidden layer were tested for each one of the networks forecasting the IMFs. First, the same number was considered for them all, ranging from more than 1000 neurons to a few tens of neurons. It was found that when a good performance was obtained, accuracy did not improve when the number of neurons increased, which fulfills what was said above regarding the number of layers: an excess of neurons tends to generate overfitting.

Then, different numbers of neurons were tested for each network forecasting different IMFs, and the higher its frequency, the higher the number of neurons needed to accurately forecast the time series. This effect sems reasonable since times series with a more complex behavior (those with the higher frequencies) should demand a higher number of neurons to be accurately forecasted, while those with lower frequencies will demand less. Thus, for the sake of simplicity, the number of neurons for the IMF with the higher frequency was fixed to 1000, and this value was reduced to 100 as the frequency decreased for the other ones, so that the last IMF, associated with the series trend, only had 100 neurons. Variations around those values were also tested, and no significant improvement in accuracy was reported.

A regularization dropout layer was also included. This process, usually carried out in RNNs such as LSTM of GRU, randomly switches off neurons in the hidden layer of the neural model to avoid overfitting, that is to say, to prevent the network from memorizing the patterns presented to it rather than learning the overall behavior defined by those patterns. In other words, when the network is overfitted, it will recognize the patterns it memorized but will not be able to identify and classify others different from those. A maximum dropout of 20% of the number of neurons was allowed for the training algorithm.

The number of neurons in the output layer is determined by the number of predictions the network must provide. Thus, its value was fixed to 24 neurons for all models.

The forecasting accuracy improvement provided by the EMD decomposition cannot be determined without a comparison with the performance provided by the same prediction model without it. Thus, the consumption time series has been directly forecasted with the LSTM and a GRU without decomposition. The number of neurons of the LSTM was fixed by trial and error as 128, compared with 64 for the GRU.

Finally, as exogenous variables other than active energy consumption were also available, the forecasting structures previously described have been also applied to forecast consumption with the four variables feeding the corresponding forecasting models. Nevertheless, as these time series have different numbers of data points, only the final 17,500 values of active and reactive consumptions were used in this case, as this is the number of data points available for the other two time series. Two scenarios were considered, one without preprocessing and the other with it, as was done for the univariate case.

The structure of the neural models was the same as in the univariate case. The number of neurons in the hidden layers when decomposition was applied remained unchanged, but it had to be increased for the case in which it was not: 256 for the LSTM and 128 for the GRU. The EMD and CEEMD decompositions were applied to each time series.

### Training–Validation Strategy: Sliding Window

When using neural networks for time series forecasting, it is usual to split the whole dataset into two subsets: one for training and the other for validation of the model, the former with a higher number of values than the latter. It is also usual to use the first data points of the series for training and the last for validation. As the learning algorithm of the neural models processes the data following their time organization, it looks reasonable to suppose that the data learned at the end of this process would be more accurately “remembered” that those processed first. Therefore, it may be assumed that the data first predicted in the validation dataset, those closer to the training set, would be more accurately forecasted that those at the end of this set. Thus, it can also be assumed that the larger the validation dataset, the less accurate the further predictions will be. This effect was observed when this division of data was applied for training and validation of the models tested in this work.

One possible way to overcome this problem is to follow the *k-fold* cross validation strategy in which the training–validation division is carried out *k* times by selecting different datasets to make up each one of those subsets, which are then independently trained–forecasted. This process has provided good results in classification problems. Nevertheless, it may not be applied to time series forecasting because it breaks the time dependency of data.

**Figure 5:**
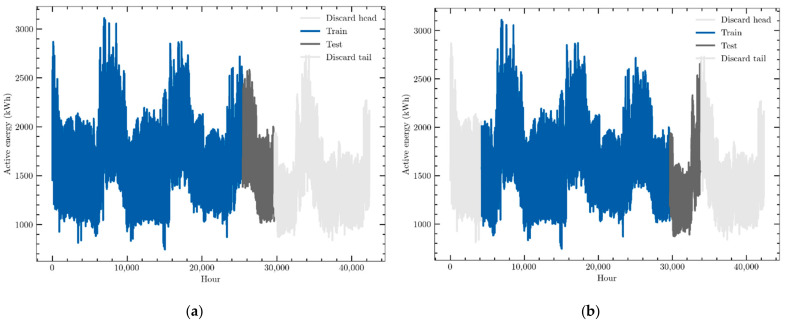

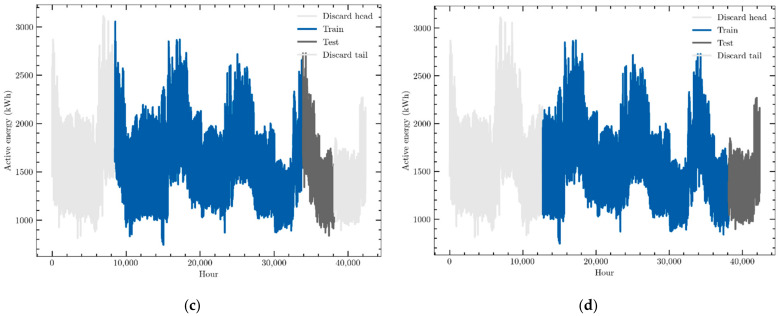
Example of the sliding window back-testing methodology; (**a**) Training set: [0–60]% data; Validation set: [60–70]% data. (**b**) Training set: [10–70]% data; Validation set: [70–80]% data. (**c**) Training set: [20–80]% data; Validation set: [80–90]% data. (**d**) Training set: [30–90]% data; Validation set: [90–100]% data.

In order to overcome these effects, a sliding window methodology has been implemented in which the size of both the training and validation datasets is fixed, although both together must be smaller than the whole time series. The process starts by training and validating the neural model with the first training–validation pair obtained from the initial data of the time series. Then, the validation dataset is added to the training one, and an equal number of data points are removed from its beginning. This will be the following training dataset, while its corresponding validation one will be made up of the following unused data. The neural model will be trained again and used for forecasting. The process is repeated until the end of the time series is reached. In this work, a division of 60–10% of the total amount of data of the time series were selected, which means that this process was repeated four times. In other words, the forecasting models have been trained and validated four times, one for each one of the four 60–10% datasets generated. This process is graphically described in Figure 5, where those four intervals are represented in each one of the four graphics. Other divisions, ranging from 40–10% to 70–10%, were tested, and very similar results were obtained with them all, although the selected one provided a slightly better performance.

Both the sliding window and the fixed division of data were tested with the models proposed in this work, and the sliding window was able to slightly improve accuracy for the furthest predictions while providing similar errors for the closest ones. Therefore, only their results are presented in this paper.

## 6. Results and Discussion

### 6.1. Evaluation Metrics

To evaluate the performance of the different models proposed, two widely used error metrics were considered: the Mean Absolute Percentage Error (MAPE) and the Root Mean Square Error (RMSE). They are defined as follows:

(15) $$MAPE=\frac{1}{N}\;\;\overset{N}{\underset{i=1}{\sum }}\left|\frac{A_{i}-F_{i}\;}{A_{i}}\right|\cdot 100,$$

(16) $$RMSE=\;\sqrt{\frac{1}{N}\overset{N}{\underset{i=1}{\sum }}{\left(A_{i}-F_{i}\right)}^{2}}$$

where *A_i_* is an actual datum, *F_i_* a forecasted one, and *N* is the total number of data points predicted.

RMSE is usually preferred to other error metrics such as the Mean Square Error (MSE) because it is measured in the same units as the original data. On the other hand, MAPE is also widely used because it is expressed in percentage terms, which makes it possible to compare performances obtained with data sets of different nature. It should be noted that MAPE can only be calculated when the data are not very close to zero, as division by a value very close to zero could provide false high errors. This was not the case for the data used in this work.

### 6.2. Forecasting Environment

As pointed out above, two types of recurrent neural networks were tested: LSTM and GRU. Each one was used to define three different forecasting structures: alone (LSTM and GRU), combined with EMD (EMD–LSTM and EMD–GRU), and combined with CEEMD (CEEMD–LSTM and CEEMD–GRU).

These forecasting models were tested in two different scenarios defined by different datasets: active energy consumption forecasted by processing only this consumption times series (univariate forecasting) and the same prediction taking into account this time series along with reactive energy consumption, temperature, and humidity (multivariate forecasting). Therefore, six different forecasting models were tested in each scenario, in other words, twelve different forecasting processes were tested. For the sake of simplicity, univariate and multivariate predictions were independently analyzed.

Simulations were carried out with the Python programing environment. The neural networks models were simulated with the high-level Keras Functional API with Tensorflow GPU 2.2 as the backend. The PyEMD package was used to perform the EMD and CEEMD decompositions. The simulations were carried out in the Spyder environment running on a workstation with an Intel Core i7-9700 CPU, 3.6 GHz with 32 GBytes of RAM and an SSD hard disc with 1 TByte. This system has also an Nvidia RTX 2070 SUPER graphics card with 8 GBytes of memory and 2560 CUDA cores. Simulations were carried out with the GPU of the graphics card to achieve shorter run times.

Different neural structures were tested for each forecasting structure. Those providing the best performances were pointed out above in Chapter 5, when describing the structure of the forecasting models. In the same way, different numbers of inputs, past data used to provide the prediction, were tested with every structure. For the univariate scenario, the best performance was achieved when 168 past data points were used, which represents one week. In contrast, only one day of past data was necessary with the multivariate scenario, which represents a total amount of 96 data points (24 data points for each variable). In all cases, one whole day of future consumptions (24 future values) were provided each time a prediction was carried out. Predictions started at 12 noon and finished at 11:00 a.m.

### 6.3. Univariate Time Series Forecasting

The future consumptions were first obtained with a univariate model in which only past data of consumption were used. Six forecasting structures were tested: LSTM and GRU alone and then combined with EMD and CEEMD. The results obtained are presented in Table 2. They were obtained processing the whole dataset of historical data of consumption. The errors achieved were organized and presented according to their time horizon, with the aim of studying how this time horizon influences the prediction accuracy. From those data, it may be seen that errors clearly increased over time. Nevertheless, when the LSTM and GRU were used alone, they experienced a slight decrease for the last data points predicted. This decrease in accuracy is logical, as it may be expected that the further the time horizon of the prediction, the lower the accuracy expected. In addition, due to the more or less cyclical behavior of consumption, it is to be expected that the accuracy will also improve when approaching a new cycle, that is to say, for time steps close to the starting hour (9:00 a.m., 10:00 a.m., and 11:00 a.m.). However, this behavior was not observed with the other four models, where the errors showed a slightly increasing trend at the beginning of the predictions and stabilized as the time horizon increased.

The overall errors were also obtained. They are shown in Table 2. The smaller ones were provided by CEEMD–GRU, although those achieved with LSTM and GRU alone and EMD–LSTM were close to it. It is worth noting that the inclusion of EMD and CEEMD in the forecasting structure did not provide a clear and significant improvement in accuracy. In fact, LSTM and GRU alone were able to achieve good performance. The only difference between the structures with or without EMD and CEEMD was that those with these preprocessing tools had more stable errors, as shown by the lower standard deviations obtained, although with the price of generating higher errors for shorter time horizons.

When comparing the performances provided by the two neural models, it may be stated that there were no significant differences between them. The same can be said when comparing EMD and its modification CEEMD.

**Figure 6:**
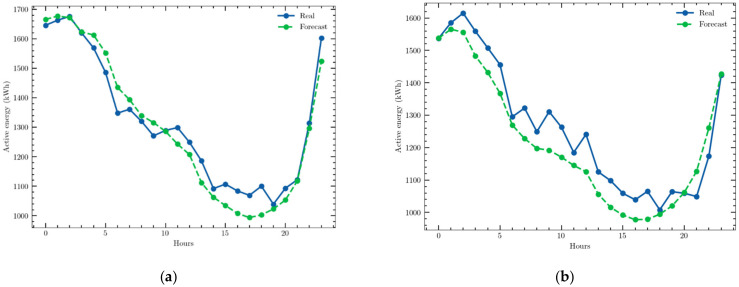
Examples of a one-day ahead prediction obtained with the univariate time series and the CEEMD–GRU structure: (**a**) 16 July 2020; (**b**) 7 January 2021.

As an example, the predictions obtained for two days with the CEEMD–GRU structure are presented in Figure 6.

### 6.4. Multivariate Time Series Forecasting

As noted in Section 4.1 above, consumption was slightly correlated with reactive consumption, temperature, and humidity. Thus, it appears reasonable to assume that the prediction accuracy could be improved if these three variables are used along with active consumption to forecast future values of this last one. In this way, those four variables were also used as inputs to the six forecasting structures used in the univariate model to predict the energy use of the next day. The results obtained are presented in Table 3, which is organized in the same way as Table 2. As stated above, only a reduced dataset of consumption, both active and reactive, was used: i.e., values coinciding with the time series of temperature and humidity.

A comparison of the results obtained with those in Table 2 shows that the performance of the six forecasting structures clearly improved. Each one was able to provide more accurate predictions than those obtained with the univariate model, for both overall and hourly errors.

The neural models without decomposition experienced a significant reduction in their forecasting errors: 7.90% to 5.29% for the LSTM and 7.86% to 5.11% for the GRU; both the overall forecasting errors and their corresponding standard deviations (1.53 to 0.88 for the LSTM and 1.54 to 0.94 for the GRU) decreased, a fact that shows that not only were the errors smaller with the multivariate model but they also had a smaller dispersion; therefore, it may be assumed that the prediction was more reliable.

When the neural networks were combined with EMD and CEEMD, the accuracy improvement was even greater: 7.80% to 3.76% for the EMD–LSTM, 8.93% to 3.51% for the CEEMD–LSTM, 8.67% to 3.68% for the EMD–GRU, and 7.68% to 3.79% for the CEEMD–GRU. Standard deviations also experienced similar reductions: 0.76 to 0.36 for the EMD–LSTM, 1.22 to 0.38 for the CEEMD–LSTM, 1.05 to 0.35 for the EMD–GRU, and 0.97 to 0.47 for the CEEMD–GRU. Again, dispersion was smaller when the neural models were combined with decomposition.

These data clearly show that the multivariate model is much more accurate than the univariate one and it should be preferred whenever exogenous data closely related to consumption are available. Therefore, it should be concluded that including data of variables correlated to consumption in the forecasting models will greatly improve the accuracy of the forecasting structures.

On the other hand, these data also show that including the time series decomposition provided by the EMD algorithm (and its refinement CEEMD) also improved the forecasting model accuracy. Although it was not able to reduce the errors in the univariate models, it provided a significant reduction for the multivariate one: 5.29% to 3.76% (EMD) and 3.71% (CEEMD) for the LSTM and 5.11% to 3.68% (EMD) and 3.79% (CEEMD) for the GRU. Thus, it may be assumed that applying these decomposition algorithms to the data to be processed by the forecasting tools clearly improves the forecasting accuracy for the multivariate models. This effect was not observed when the univariate time series data were used.

When the errors were analyzed considering their hourly evolution, it was concluded that both LSTM and GRU alone followed the same behavior as for the univariate case: the error increased as the time step increased for the first predictions but changed to a decreasing trend from the 12th prediction onwards. When decomposition was included, the behavior was again similar to that of the univariate case: the errors had a slight increasing behavior, although the EMD–LSTM showed a slight decrease at the latest time steps.

The best performance was provided by the CEEMD–LSTM structure although the errors it provided were only slightly lower that those achieved by the other three structures, including decomposition. Therefore, it can be concluded that both LSTM and GRU are able to provide accurate predictions, which are further improved when EMD (or CEEMD) is applied to the time series. Regarding the decomposition algorithm, the results achieved showed that the modification of the basic EMD, the CEEMD, did not provide a significant improvement of the prediction accuracy. In fact, although accuracy slightly improved with the LSTM, it slightly decreased with the GRU.

**Figure 7:**
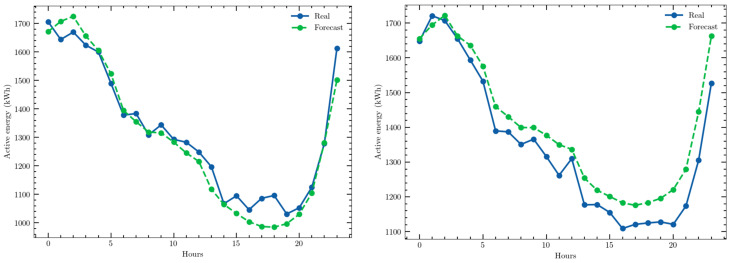
Examples of a one-day ahead prediction with the multivariate time series and CEEMD–LSTM model: 19 April 2021; 1 July 2021.

The predictions obtained for two different days with the CEEMD–LSTM structure are shown in Figure 7 as examples of the high accuracy achieved.

### 6.5. Discussion

It is difficult to compare the performance of different forecasting tools applied to different time series. Even those with similar behavior, such as different kinds of buildings or different companies working in the same sector, usually have different consumption profiles that provide consumption time series with a more or less similar behavior but with specific features that make them significantly different. Most of the models proposed in literature are adapted to the time series they are to process to provide the best possible accuracy. Nevertheless, it is not guaranteed that these models will also provide such good results when applied to time series other than those they were designed to process. The performances achieved could be good but usually will not be the best. On the hand, comparisons should be carried out only when a percentual figure of merit is used, such as MAPE, because other error measurements will be related to the nature and value range of the time series forecasted. Thus, the results achieved in this work may only be compared with a few studies. In [10], hourly consumption of a household was predicted with a multivariate model. The best result was provided by a seasonal ARIMA: an MAPE of 1.162%. It was able to outperform an LSTM and an XGBoost. In this work, only consumptions of five days were used for training and forecasting, and one only day was predicted, that is to say, only one prediction was carried out. In [8], daily and monthly consumptions of 28 commercial building were analyzed and their consumptions predicted with five forecasting tools. The best performance for the daily predictions was provided by an LSTM with an MAPE of 8.97%, although most of them were higher than 10%. In [51], consumptions of three different building were forecasted. The predictions of the following 24 hourly data were provided by 24 GRUs with an attention structure (encoder–decoder network) that allowed the predictor to focus on the most relevant input variables (a multivariate structure was used). The performance of this structure was compared with others (Random Forest, LSTM, Deep Neural Network) and it was able to provide a better MAPE: 6.09%. In [11], consumptions of several building in a university were provided. A Bidirectional LSTM Sequence-to-Sequence structure combined with an attention mechanism (S2SATT-BiLSTM) provided predictions for the following day at 15-min intervals (96 future data points). Its performance was compared with that provided by other models (GRU, Multilayer Perceptron, Gradient Boosting Machine, XGBoost, Random Forest), and it outperformed them. The best MAPE obtained was 4.42%.

When comparing the results presented in those works with those achieved by the models proposed in the present one, it can be seen that the CEEMD–LSTM (which provided the best one: an MAPE of 3.51%) clearly outperformed the models proposed in those works (in [10], a better MAPE was obtained, but only for a single prediction, a result that could be considered of little significance). Nevertheless, as pointed out above, these comparisons may only be performed from a relative point of view. The models proposed in the works above were applied to different time series describing different consumers. They all are small or medium ones but are very different with different consumption profiles. Therefore, the only thing that can be stated with certainty is that the models proposed in those works were able to provide accurate predictions for the short-term consumption time series they have forecasted.

All of the works mentioned above used a multivariate structure (weather variables, type of day, type of the elements consuming electricity) to provide their predictions. Some works [7,10] have showed that this structure outperforms the univariate one. The results achieved in the present work clearly support this fact.

It should be pointed out that only one of those works use simple models (ref. [10], with simple Seasonal ARIMA, LSTM and XGBoost); however, the small number of simulations performed (one day) did not result in significant predictions. Other researchers used hybrid models that comprise several algorithms to improve the performance of basic forecasting models (Random Forest, LSTM, GRU). They were able to provide better accuracies than those simple models. In some cases, very complex forecasting structures were defined. In the present work, hybrid structures were also proposed, although they are not very complex: a single Recurrent Neural Network along with a preprocessing algorithm. This fact can be assumed a significant contribution of this work, as a relatively simple hybrid forecasting model has been able to provide accurate results that, from a relative point of view, can outperform other forecasting tools with more complex structures. Both the neural models and the EMD are easily programmed since their basic structure may be implemented directly from widely used programming environments such as that used in this work, Python, or any other that can be found on the market (R, Matlab).

Finally, run times of the training process of several of the simulated models are provided in Table 4. They were related to the number of neurons each neural model has. Only the multivariate scenario is presented as it provided the best performance. The corresponding overall errors for each model are also shown. Run times for the validation stage have not been provided because they were insignificant (lower that 1 s in all cases) when compared with those of the training processes. It can be seen that those times are much longer for the hybrid models because they require ten neural networks to be trained. Nevertheless, they must not be considered as excessively long since a high number of neurons are considered and, in the case of the hybrid models, ten neural networks need to be trained.

## 7. Conclusions

In this work, a hybrid forecasting structure is proposed, which is made up of a Recurrent Neural Network and a preprocessing algorithm, to predict the hourly consumption of a whole day for a hospital.

Two forecasting scenarios were tested: a univariate model that only used the consumption time series to provide the predictions and a multivariate one that also included other three exogenous variable: reactive consumption, humidity, and temperature. The multivariate model provided significantly more accurate predictions in all cases tested. Thus, it may be stated that exogenous variables should be included in the forecasting models whenever they are available.

Two neural networks were used: LSTM and GRU. They were able to provide similar performances, as no significant differences were recorded.

However, the inclusion of a preprocessing stage carried out with EMD and CEEMD significantly improved the accuracy of the multivariate models (those that provide the most accurate predictions). They were able to significantly reduce the overall errors in all cases, and, what could be considered more significant, they were able to provide a very stable behavior of errors, which showed a slight increasing trend that was almost constant for the second half of the predictions. It is worth noting that both EMD and CEEMD were able to improve accuracy, providing reliable predictions both with LSTM and GRU, with very small differences in the errors achieved.

Thus, it may be concluded that including exogenous variables in the forecasting models greatly improves their accuracy. In fact, when it is not, preprocessing does not affect accuracy. Only when they were used were EMD and CEEMD able to significantly improve the forecasting tool performance. In this scenario, the hybrid models represent a better choice than the single forecasting models.

The forecasting tools developed were able to provide accurate and reliable predictions of hourly consumptions in a hospital. The nature of its activity means that its time series have a variable seasonal and hourly behavior, but without extreme peaks. In fact, the COVID-19 epidemy experienced all over the world in 2020 and 2021 had no significantly modified consumption profiles. Thus, further works should be carried out to test these tools with consumers different from that analyzed here, with more complex consumption profiles and with extreme demands that could be influenced by social or political events.

In any case, it may be stated that the models proposed in this work may be useful and valuable for companies managing electricity purchases for themselves or for other customers with a more or less stable consumption profile. Those tools have proved to provide accurate predictions of hourly consumptions for a whole day, so that those companies can look for the best prices in the electric market, which will enable them to make the most profitable offers. Therefore, these tools can help these companies plan their purchasing polices to try to reduce their electricity bills. On the other hand, it is worth noting that implementing the forecasting structures presented in this work will be free of charge for companies interested in using them, as they were developed with freeware software, and there are many free online tools to effectively program those programmed in this work and any other Artificial Intelligence model or data processing algorithm, which greatly facilitates their programing.

This work may be assumed to be a first step in the study of short-term predictions of electricity consumption in small–medium companies. Further works should be developed to apply the forecasting tools proposed here to different time series representing different consumption profiles to test their generalization capabilities. Thus, different values for the hyperparameters defining their structures should be tested to better adapt these tools to the specific time series they are forecasting, which represent the consumption profiles of different consumers with different electric energy needs.

In addition, other neural models, such as the classical Multilayer Perceptron (MLP), and other preprocessing tools, such as the wavelet transform, could also be tested. All of them should be applied to different consumption time series from different consumers to find out the best prediction structure or, at least, to identify which of them provides the best performance for each time series.

## Figures and Tables

**Table 1:** Correlation matrix of the four variables used to predict consumption.

	Active Energy	Reactive Energy	Temperature	Humidity
**Active Energy**	1	0.58	0.67	−0.55
**Reactive Energy**	0.58	1	0.39	−0.32
**Temperature**	0.67	0.39	1	−0.83
**Humidity**	−0.55	−0.32	−0.83	1

**Table 2:** Prediction errors obtained with the univariate model.

Hours	LSTM		GRU		EMD–LSTM		EMD–GRU		CEEM-LSTM		CEEMD–GRU	
Ahead	RMSE	MAPE	RMSE	MAPE	RMSE	MAPE	RMSE	MAPE	RMSE	MAPE	RMSE	MAPE
1	69.42	3.49	69.53	3.60	106.85	6.27	112.66	6.89	104.76	6.27	94.02	5.67
2	97.24	4.82	93.91	4.73	111.63	6.14	119.68	6.98	110.52	6.33	97.11	5.48
3	119.56	5.80	116.50	5.64	125.51	6.66	128.12	7.15	122.53	6.86	109.98	6.01
4	137.90	6.61	135.58	6.38	135.31	6.92	137.77	7.42	130.80	7.19	121.18	6.41
5	154.67	7.31	153.48	7.18	144.56	7.25	145.52	7.61	140.77	7.66	128.60	6.58
6	167.24	7.91	166.15	7.70	151.24	7.49	151.45	7.72	148.90	8.08	140.58	7.16
7	175.19	8.38	173.31	8.01	152.77	7.54	155.85	7.89	154.80	8.40	146.93	7.48
8	178.11	8.66	176.53	8.32	151.75	7.41	158.87	8.06	158.51	8.62	150.03	7.57
9	178.63	8.80	176.53	8.48	152.70	7.52	160.49	8.23	164.65	9.00	153.98	7.76
10	178.71	8.95	176.52	8.67	154.56	7.60	160.38	8.25	169.22	9.29	156.35	7.78
11	179.73	9.17	177.44	8.95	156.97	7.64	162.72	8.33	173.60	9.55	159.01	7.80
12	179.50	9.35	177.43	9.20	161.12	7.70	168.04	8.55	176.56	9.61	161.69	7.81
13	180.23	9.49	178.59	9.38	168.32	7.93	173.91	8.73	181.56	9.81	168.17	8.15
14	179.63	9.49	178.81	9.47	174.55	8.10	179.56	8.93	184.19	9.84	171.19	8.27
15	176.61	9.36	177.02	9.44	180.36	8.21	186.42	9.18	188.09	9.89	173.41	8.29
16	173.04	9.11	175.40	9.31	185.50	8.39	193.16	9.40	191.02	9.87	180.15	8.54
17	169.15	8.91	174.58	9.17	187.40	8.51	199.68	9.67	193.14	9.82	181.39	8.55
18	165.71	8.69	173.18	8.99	188.03	8.54	204.25	9.89	194.14	9.70	184.97	8.62
19	161.09	8.36	166.95	8.62	190.05	8.63	206.01	9.91	194.83	9.65	186.10	8.51
20	155.07	8.01	157.97	8.14	190.60	8.59	208.22	10.00	195.12	9.65	187.34	8.54
21	147.68	7.56	148.73	7.65	195.03	8.72	208.29	9.93	196.03	9.71	186.85	8.52
22	140.75	7.19	142.09	7.32	194.34	8.60	208.17	9.87	196.82	9.77	185.40	8.40
23	138.15	7.03	139.43	7.12	193.49	8.43	207.63	9.77	198.40	9.88	181.90	8.23
24	140.45	7.22	141.06	7.18	193.51	8.36	205.86	9.64	198.82	9.96	180.76	8.25
**Mean**	155.98	7.90	156.11	7.86	164.42	7.80	172.61	8.67	169.49	8.93	157.80	7.68
**Std**	28.55	1.53	28.94	1.54	26.70	0.76	30.44	1.05	29.36	1.22	29.08	0.97

**Table 3:** Prediction errors obtained with the multivariate model (active energy, reactive energy, temperature, and humidity).

Hours	LSTM		GRU		EMD–LSTM		EMD–GRU		CEEMD–LSTM		CEEMD–GRU	
Ahead	RMSE	MAPE	RMSE	MAPE	RMSE	MAPE	RMSE	MAPE	RMSE	MAPE	RMSE	MAPE
1	54.83	3.22	48.74	2.93	40.29	2.60	40.14	2.58	37.66	2.42	38.66	2.44
2	71.65	3.88	60.79	3.38	46.62	2.96	45.75	2.93	42.82	2.72	43.92	2.80
3	80.69	4.39	72.43	3.96	52.14	3.31	50.95	3.23	47.20	2.97	48.40	3.06
4	86.52	4.81	84.14	4.68	55.06	3.50	53.91	3.43	49.97	3.22	52.55	3.30
5	90.03	5.04	90.72	5.05	56.75	3.62	55.98	3.55	51.71	3.36	54.26	3.43
6	93.37	5.32	92.22	5.20	57.80	3.66	56.76	3.60	52.99	3.43	55.79	3.53
7	96.37	5.65	95.16	5.48	58.48	3.74	57.23	3.65	53.66	3.52	56.40	3.58
8	98.52	5.85	97.51	5.76	59.98	3.81	57.43	3.63	54.80	3.56	58.62	3.73
9	100.46	6.10	98.23	6.00	62.21	3.95	59.49	3.71	56.17	3.60	61.97	3.95
10	100.61	6.22	98.46	6.12	64.16	4.02	60.83	3.81	58.37	3.67	63.90	4.03
11	101.52	6.36	98.36	6.22	65.88	4.11	61.30	3.81	59.98	3.69	64.69	4.03
12	100.87	6.37	97.08	6.20	64.91	4.07	63.10	3.86	59.56	3.67	64.75	4.04
13	99.39	6.33	95.51	6.19	64.92	4.11	62.44	3.80	58.66	3.62	64.56	4.03
14	97.44	6.24	93.49	6.15	63.93	4.03	63.32	3.87	57.05	3.52	64.85	4.02
15	94.16	6.01	90.80	5.91	62.53	3.94	60.50	3.73	55.38	3.48	65.28	4.08
16	92.69	5.88	88.76	5.70	62.10	3.95	60.19	3.78	54.54	3.46	65.64	4.11
17	90.98	5.71	87.83	5.54	61.21	3.94	58.52	3.72	54.69	3.52	65.82	4.14
18	88.92	5.54	85.75	5.33	60.09	3.87	58.59	3.77	55.08	3.55	66.11	4.16
19	86.35	5.30	82.27	5.04	59.61	3.84	59.12	3.84	56.11	3.62	65.06	4.13
20	83.63	5.06	78.16	4.75	58.85	3.82	59.79	3.86	57.31	3.70	61.85	3.94
21	78.55	4.71	73.38	4.47	58.34	3.82	60.23	3.94	58.80	3.81	60.88	3.93
22	72.47	4.24	68.99	4.16	58.13	3.83	60.96	3.98	61.08	3.96	62.39	4.01
23	74.18	4.30	69.94	4.14	58.56	3.80	63.10	4.11	63.30	4.06	64.58	4.19
24	77.10	4.50	74.32	4.37	59.55	3.87	65.12	4.21	64.48	4.09	66.39	4.24
**Mean**	87.97	5.29	84.29	5.11	58.84	3.76	58.11	3.68	55.06	3.51	59.89	3.79
**Std**	11.89	0.88	13.35	0.94	5.83	0.36	5.69	0.35	6.06	0.38	7.53	0.47

**Table 4:** Run times and overall prediction errors of three configurations (number of neurons of the neural networks) of the forecasting models. Only one preprocessing algorithm of the hybrid models is presented that provided the lower errors because the differences in performance between EMD and CEEMD were very small. The best performances are shown in boldface type.

Model	No. of Hidden Neurons	Elapsed Training Time (s)	Overall MAPE (%)
	128	139	5.43
LSTM	256	202	**5.29**
	512	268	5.37
	32	43	5.36
GRU	128	98	**5.11**
	256	171	5.20
	100 per IMF	1251	3.87
CEEMD–LSTM	1000 per IMF	3033	4.09
	1000-100 decay	2197	**3.51**
	100 per IMF	915	3.88
EMD–GRU	1000 per IMF	2623	4.19
	1000-100 decay	1894	**3.68**

## Data Availability

Not applicable.
